# New Procedure to Maintain Fecal Microbiota in a Dry Matrix Ready to Encapsulate

**DOI:** 10.3389/fcimb.2022.899257

**Published:** 2022-06-10

**Authors:** Andrea Aira, Elisa Rubio, Andrea Ruiz, Andrea Vergara, Climent Casals-Pascual, Verónica Rico, Josep Maria Suñé-Negre, Alex Soriano

**Affiliations:** ^1^Department of Infectious Diseases, Hospital Clinic of Barcelona, Barcelona, Spain; ^2^University of Barcelona, Barcelona, Spain; ^3^Department of Clinical Microbiology, Hospital Clinic of Barcelona, Barcelona, Spain; ^4^Barcelona Institute for Global Health (ISGlobal), Barcelona, Spain; ^5^Institut d’Investigacions Biomèdiques August Pi i Sunyer (IDIBAPS), Barcelona, Spain; ^6^Faculty of Pharmacy, University of Barcelona, Barcelona, Spain

**Keywords:** FMT, gut microbiota, adsorbate, lyophilization, capsules

## Abstract

Fecal microbiota transplantation (FMT) is one of the recommended treatments for recurrent *Clostridioides difficile* infection, but endoscopy and available oral formulations still have several limitations in their preparation, storage, and administration. The need for a viable oral formulation that facilitates the implementation of this highly effective therapy in different settings has led us to test the microcrystalline cellulose particles as an adsorbent of concentrated filtered fresh feces in comparison to lyophilized feces. This free-flowing material can provide protection to bacteria and results in a dried product able to maintain the viability of the microbiota for a long time. Adsorbate formulation showed a stabilizing effect in gut microbiota, maintaining bacteria viability and preserving its diversity, and is a competitive option for lyophilized capsules.

## Introduction

The use of fecal microbiota transplantation (FMT) to restore a recipient’s gut microbial composition is one of the recommended treatments for recurrent *Clostridioides difficile* infection (Johnson et al., 2021; van Prehn et al., 2021). It is usually performed using 50 g of fresh feces from healthy donors, filtrated, and administered *via* the lower or upper gastrointestinal route such as endoscopy or colonoscopy or after additional processing to be administered as oral capsules with high success rates (McDonald et al., 2018; Mullish et al., 2018; Cammarota et al., 2019). Current efforts are focused on the development of new oral formulations that facilitate the implementation of this highly effective therapy in different settings. The most widely tested options include direct encapsulation of the filtered feces (fresh or previously frozen) or processing the feces to obtain a dried product containing viable microbiota (i.e., lyophilization) that is easy to encapsulate (Reigadas et al., 2018; Fadda, 2020). These options have shown good tolerability and high success rates, and consequently have increased the use of FMT, but still have several limitations in its preparation, storage, and administration (Youngster et al., 2016; Staley et al., 2017; Reigadas et al., 2020).

There is a need for a simple and cheaper process to obtain an effective dried product from feces while maintaining microbiota viability, reducing the odor and the number of capsules per 50 g of feces, and being easy to store and transport. This has led to search other processes and materials including the use of microcrystalline cellulose particles, which are a free-flowing material that does not form particle agglomerates and can provide protection to bacteria. This material is used to protect and encapsulate probiotics and is one of the most useful tablet and capsule diluent with adsorbent properties (Kocherbitov et al., 2008; Nofrerias et al., 2019; Sánchez-Portilla et al., 2020; Sheskey et al., 2020). We hypothesized that the use of microcrystalline cellulose particles could act as an adsorbent of concentrated filtered fresh feces to obtain a dried product able to maintain the microbiota viability for a long time at room temperature or at 4°C.

Our aim was to evaluate the use of microcrystalline cellulose in combination with an excipient as an adsorbent to obtain a dried product from feces in comparison to a lyophilized formulation. We analyzed the different formulations’ capacity to preserve bacterial viability and diversity over time.

## Materials and Methods

### Sample Processing

Feces were collected from healthy donors in a specific recipient for this purpose (Fecotainer^®^, AT Medical B.V., Netherlands) with a BD Gaspak™ EZ anaerobe system (Becton Dickinson and Company, USA) attached to the lid to maintain anaerobiosis. The samples, with a minimum of 50 g, were brought to the lab, maintained at 4°C, and processed as a pool within 4–6 h from collection. Pooled feces were transferred to a stomacher bag, sterile saline was added (10:1), and the mix was homogenate in Stomacher 400 circulator (Seward Ltd., United Kingdom) for 1 min at 230 rpm. Then, the pool was transferred to falcon tubes; 10% of pure glycerol was added and frozen at −80°C.

When required, samples were thawed overnight at 4°C and 20% of pure glycerol was added. They were centrifuged at 4°C in a Heraeus Megafuge 16R Centrifuge (Thermo Fisher Scientific Inc., USA) for 20 min at 400 g, with slow deceleration to remove sample debris. The supernatant was filtered with a conventional sieve to eliminate possible detritus, transferred into high resistant tubes, and centrifuged at 4°C for 30 min at 10,000 *g* (Sorvall Evolution RC Centrifuge, Thermo Fisher Scientific Inc., USA). The pellet with the concentrated microbiota was recovered with a spatula after decantation of the supernatant avoiding any remaining liquid.

For lyophilized capsules, the pellet was disposed into empty petri dishes and frozen at −80°C for at least 1 h. Then, they were introduced in the lyophilizator Telstar Liomega 3 (LI1) (Telstar, Spain) with a starting shelf temperature of −40°C and secondary drying for 10 h at +25°C. All steps were done under 90 to 150 mbar vacuum, and the whole process took 48 h. The lyophilized product was manually encapsulated due to its viscosity and morphological characteristics into 00 acid-resistant capsules (Capsugel^®^, Lonza, Switzerland) obtaining between 3 and 5 capsules per treatment (from 50 g of feces).

For the new formulation capsules (patent application WO2020212297A1), named adsorbate capsules, the pellet volume equivalent to 50 g of feces was mixed manually in a mortar with microcrystalline cellulose Vivapur-101^®^ (JRS Pharma, Germany) and magnesium stearate in a proportion of 50:1 until a final homogeneous powder-like product is obtained. Vivapur-101^®^ acted in the sample as a water adsorbent, and magnesium stearate was added to facilitate the flowability of the product into capsules. The powder was kept overnight at 4°C in a fridge surrounded by Silica gel plaques to reduce the humidity around the mixture. Then, the powder was encapsulated with a semi-automated encapsulator FagronLAB™ FG (Fagron Iberica, Spain) into 00 acid-resistant capsules, obtaining between 14 and 20 capsules per treatment (from 50 g of feces). Both lyophilized and adsorbate capsules were kept at 4°C with Silica gel bags and labeled for its traceability until analysis.

### Bacterial Viability in Lyophilized and Adsorbate Capsules

**Figure 1:**
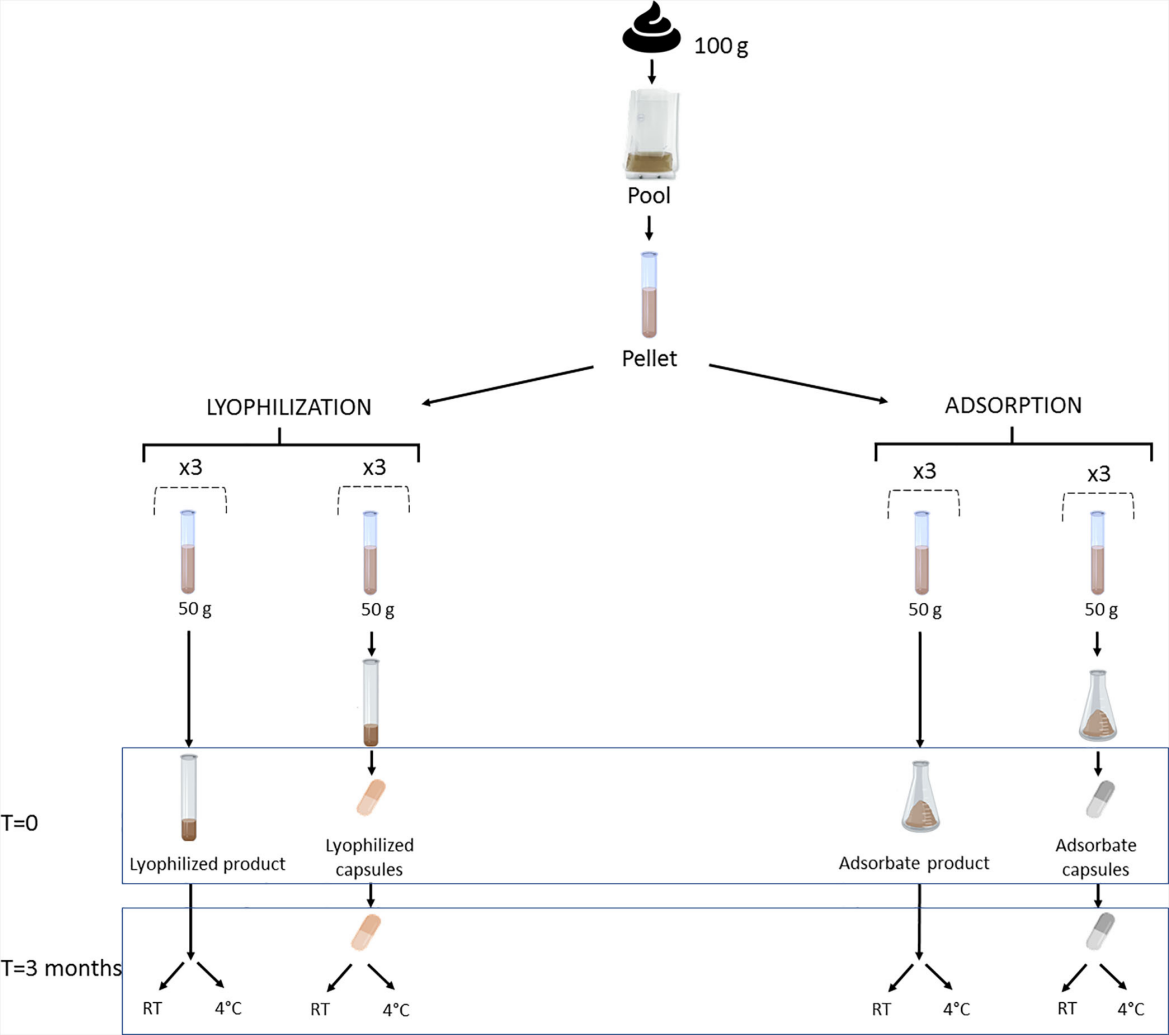
Experiment design to compare bacterial viability in lyophilized and adsorbate capsules. Three replicates of each processing formulation were encapsulated, and three more were kept as lyophilized and adsorbate products. The analysis was performed in each aliquot at time point 0 and 3 months after being stored at room temperature (RT) and at 4°C. Created with BioRender.com.

We processed a 600-g pool of feces to obtain the pellet as previously described and separated it into two parts for lyophilization and adsorption experiments. Each part was divided into six identical replicates containing an equivalent of 50 g of feces each. Three replicates from each experiment (lyophilization and adsorption) were encapsulated to test the evolution of the product into the capsules (**Figure 1**).

We tested bacterial viability using two different methods: (1) flow cytometry with LIVE/DEAD™ Baclight™ Bacterial Viability and Counting Kit (Thermo Fisher Scientific, USA), and (2) quantitative bacterial culture of sample dilutions 1:10000, 1:1000, and 1:100 in Columbia Agar with 5% sheep blood (BD GmbH, Germany) incubated overnight at 37°C in aerobic and anaerobic conditions. The cytometer used was BD FACSCantoII (BD Biosciences, USA) and software was BD FACSDiva 8.0 following the manufacturer’s instructions. Measurements were performed in the original pool and capsules from each replicate at time 0 (just after preparation) and 3 months after in duplicate, keeping samples at room temperature (RT) and at 4°C.

For flow cytometry analysis, encapsulated and non-encapsulated lyophilized and adsorbate aliquots were diluted to 1:10000 using 0.9% NaCl solution and vortexed vigorously. SYTO9 (1:1, 0.1 µl), propidium iodide (1:1, 0.1 µl), and microspheres (1:2, 10 µl) were added, for a final volume of 250 µl. The concentration of live bacteria was determined following the protocol equation (Molecular Probes Inc., 2004).

### Bacterial Stability With or Without Magnesium Stearate in Adsorbate Formulation

In order to evaluate the impact of magnesium stearate in adsorbate formulation, three feces from healthy donors were obtained and processed in parallel as previously described. Pellets from each fecal sample were separated into two identical aliquots representing 50 g of feces. One part was mixed with Vivapur-101^®^ (named V capsules) whereas the other was mixed with Vivapur-101^®^ and magnesium stearate (named VMs capsules). Both products were semi-automatically encapsulated into 00 acid-resistant capsules (**Supplementary Figure S1**).

We tested bacterial viability in the initial samples and capsules using flow cytometry and bacterial culture at time 0, and 3 and 6 months after storage at 4°C.

### Analysis of Microbial Composition

Samples from all experiments were stored at −80°C at different time points until they are processed for microbial analysis. We determined taxonomical composition and alpha diversity in order to check product stability in terms of microbial composition.

DNA was extracted using the PureLinkTM Microbiome DNA Purification Kit (Invitrogen, USA). The 16S rRNA gene V3–V4 region was amplified and sequenced on an Illumina MiSeq platform (2 × 300 bp) following the Illumina 16S Metagenomic Sequencing Library Preparation protocol using KAPA HiFi HotSart polymerase (Roche, Switzerland). The obtained sequences were filtered and demultiplexed using the DADA2 pipeline. Diversity metrics, compositional, and statistical analyses were performed using QIIME (QIIME 2 version 2020.2) and R version 3.4.4. Taxonomy was assigned using Silva version 132. Samples with <1,000 sequence reads were removed. Singletons and features with a relative frequency <0.01% were also removed. Finally, samples were rarefied to 4,300 read sequencing depth for alpha diversity, beta diversity, and compositional calculations.

For microbial diversity analysis, evenness (Pielou index) and Faith indices were calculated and clustering analysis was performed using Bray–Curtis dissimilarity distances at the feature level.

### Macroscopic and Humidity Analysis

Visual inspection of capsules was evaluated at each time point, including size measurement and macroscopic aspect of the capsules. The humidity of the encapsulated adsorbate product was evaluated after storage at 4°C with or without Silica gel, using the Karl-Fischer method (899 coulometer, Metrohm, Switzerland) according to Pharmacopoeia 9.4., section 2.5.12. The humidity was tested in three capsules individually for each condition using Hydranal-Coulomat AG (Thermo Fisher Scientific, USA) as a reactive. From the content of each capsule, 100 mg was taken as aliquot and was analyzed with an agitation parameter rate of 10.

### Scanning Electron Microscope Observation

For scanning microscope analysis, the content of an adsorbate capsule was fixed in a solution consisting of 2.5% glutaraldehyde in 0.1 M phosphate buffer (pH 7.4), post-fixed in osmium tetroxide (1%) in the same phosphate buffer, dehydrated in graded alcohol, and dried for critical point drying using Emitech K850. Samples were covered with a carbon thin film in order to improve their electrical conductivity. The samples were observed with a Jeol JSM-7001F (Jeol, Japan) operated at 15 kV. We used a preparation of mixed VMs excipients as control.

### Statistical Analysis

Continuous variables were analyzed using a two-sided *t*-test using R 3.6.2 version and considering a *p* < 0.05 to be statistically significant. Graphs were obtained with GraphPad Prism 9.2.0 and R 3.6.2.

## Results

**Figure 2:**
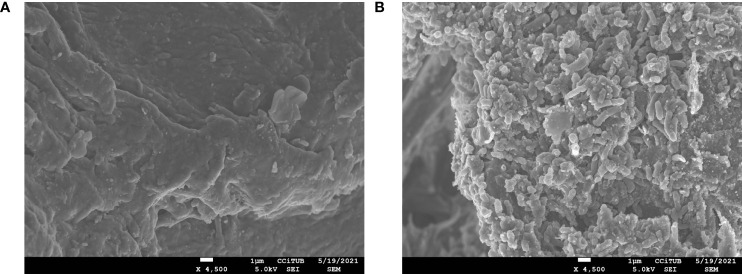
Image from scanning electronic microscope of Vivapur-101^®^ + magnesium stearate (×4,500) before **(A)** and after **(B)** mixing with concentrated filtered feces. In **(A)**, we observed the surface of Vivapur-101^®^ microfibrils, and in **(B)**, the surface was completely covered by a film of bacteria with different morphologies.

The adsorbate product presented a homogenous powder-like appearance that was easy to manipulate in contrast to the viscosity of the lyophilized product. By using a scanning electron microscope, we observed that, in the adsorbate product, the bacteria were homogenously attached to Vivapur-101^®^ microfibrils (**Figure 2**).

### Comparison of Bacterial Viability in Lyophilized and Adsorbate Capsules

**Figure 3:**
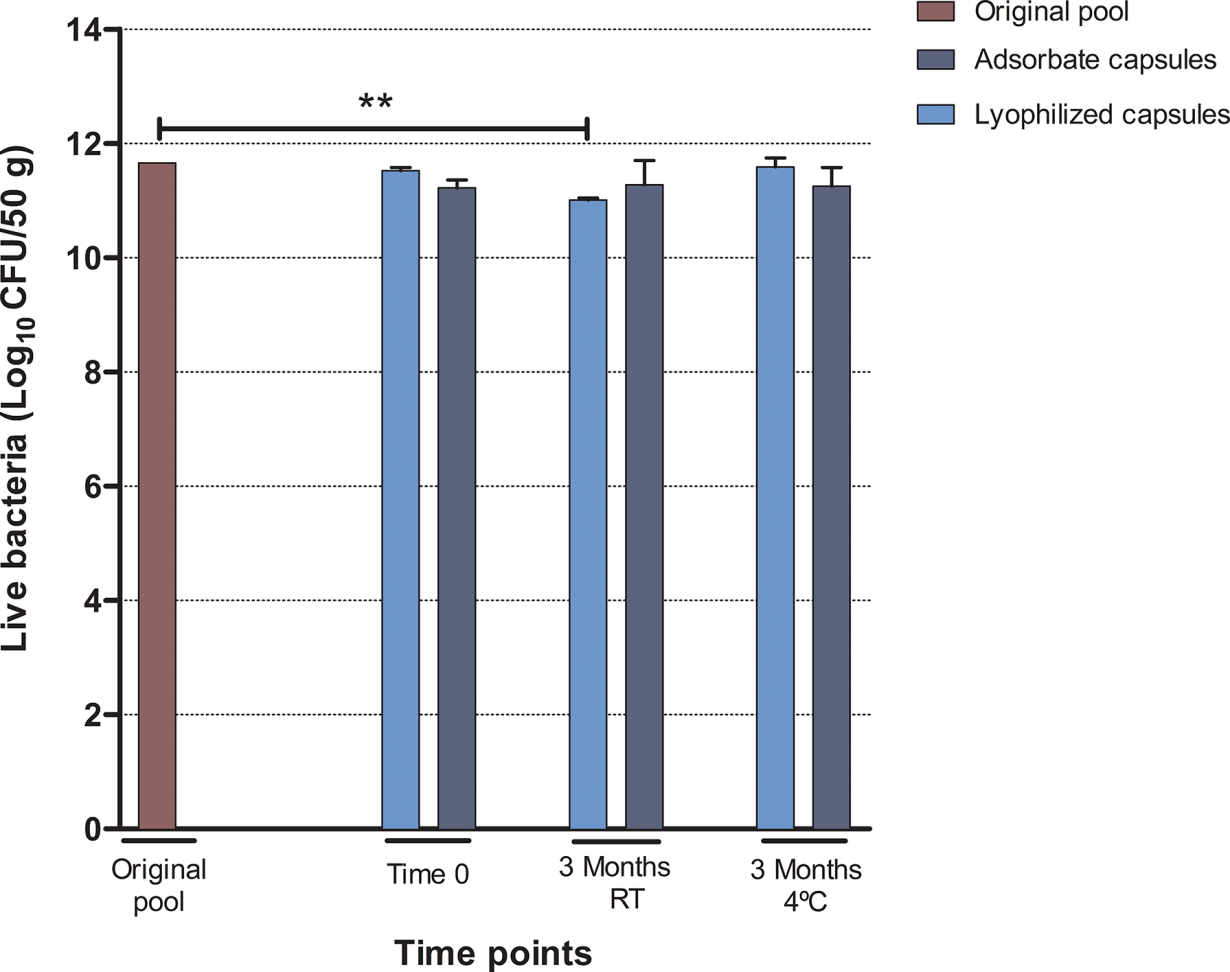
Results from flow cytometry analysis corresponding to the number of viable bacteria expressed as the mean of Log_10_ CFU/50 g of feces (SD) in each step and after 3 months of storage at room temperature (RT) or 4°C. Statistical significance **<0.005.

The bacterial viability results in pool and both capsule formulations studied by flow cytometry are depicted in **Table 1** and **Figure 3**. The comparisons of bacterial viability in formulations were made using the original pool as control. The bacterial viability in the lyophilized formulation non-significantly decreased just after preparation (*p* = 0.05) but differed significantly after 3 months of storage at room temperature (*p* = 0.0009). This was not observed when stored at 4°C (*p* = 0.52), showing a greater loss in bacterial viability when lyophilized capsules were stored at RT compared to 4°C (*p* = 0.02).

**Table 1:** Results from flow cytometry of lyophilized and adsorbate capsules.

		Pool	Lyophilized capsules	*p*	Adsorbate capsules	*p*
*T* = 0		11.66	11.52 (0.06)	*p* = 0.05*	11.22 (0.14)	*p* = 0.14**
*T* = 3 months	RT	NA	11.01 (0.03)	*p* = 0.0009*	11.28 (0.43)	*p* = 0.26**
	4°C	NA	11.59 (0.16)	*p* = 0.51*	11.25 (0.33)	*p* = 0.16**

*Compared to pool results. **Compared to pool results.

Data are presented as the mean of live bacteria (Log_10_ CFU/50 g of feces) from replicates and standard deviation (SD).

NA, Non Applicable.

On the other hand, no significant changes on bacterial viability were observed in the adsorbate formulation just after preparation (*p* = 0.14), after 3 months of storage at room temperature (*p* = 0.26), or at 4°C (*p* = 0.16) in comparison to the original pool.

When comparing the two formulations, no significant differences were found at time point 0 (*p* = 0.17), 3 months at RT (*p* = 0.39), or 3 months at 4°C (*p* = 0.21). The results from quantitative culture supported the results from flow cytometry (**Supplementary Data 1**).

**Figure 4:**
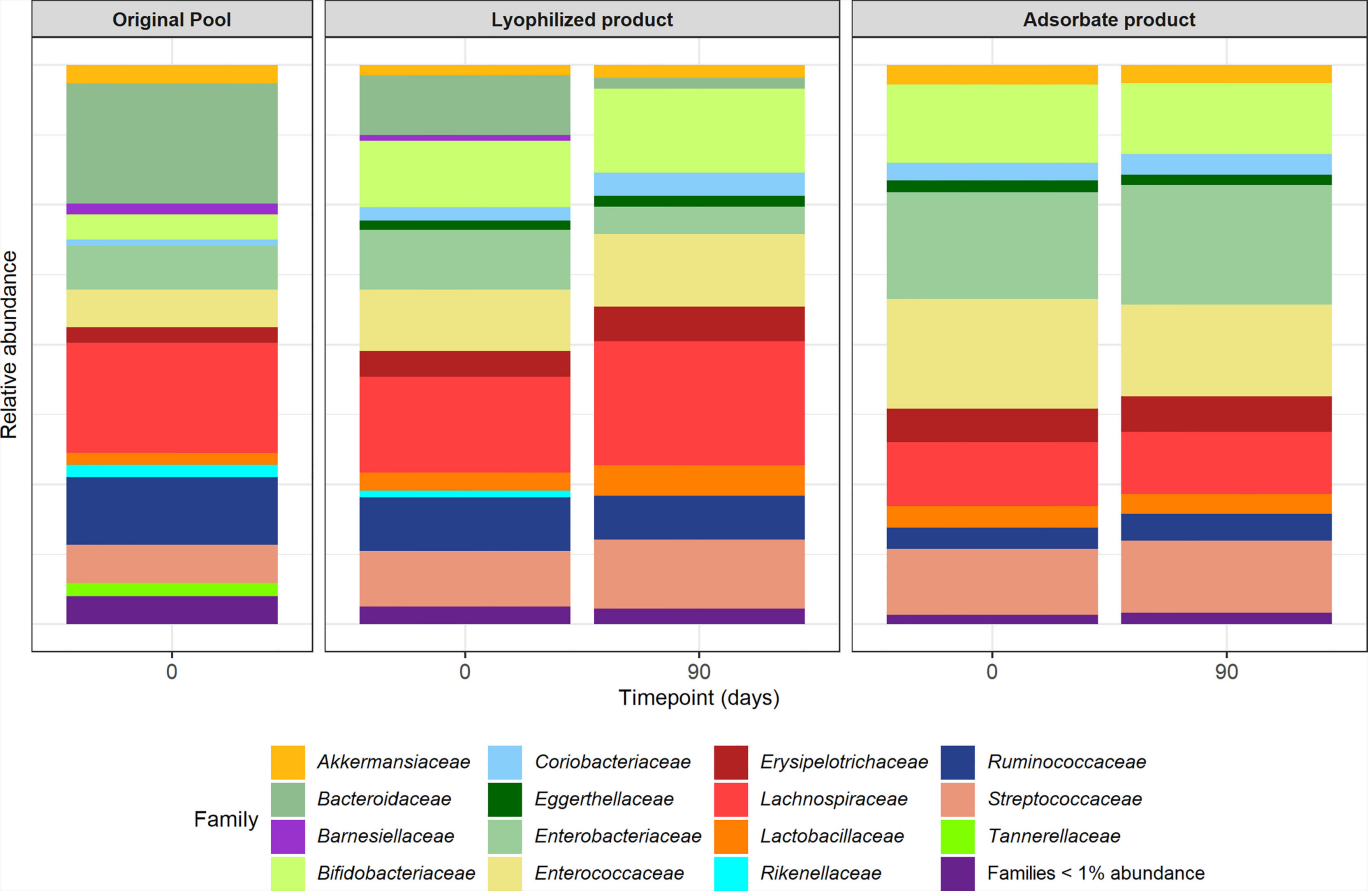
Relative abundances of bacterial taxonomical composition of original pool and lyophilized and adsorbate capsules at time point 0 and 3 months.

From genetic analysis, the samples from both formulations corresponding to 3 months after storage at RT could not be recovered after DNA extraction protocol and sequencing was performed for lyophilized and adsorbate capsules at time point 0 and after 3 months at 4°C. The relative abundances at the family level are shown in **Figure 4**. From the original pool to capsules, there was a reduction in the relative abundance of *Bacteroidaceae*, which was more pronounced in the adsorbate formulation compared to the lyophilized formulation. We also observed an overrepresentation of some families such as *Enterococcaceae* and *Streptococcaceae* in both types of formulations. However, the relative abundances of families such as *Bifidobacteriaceae*, *Lactobacillaceae*, and *Ruminococcaceae* were conserved after lyophilization and adsorbate processing and storage for 3 months.

The lyophilized product showed a better maintenance of the relative abundances at the family level compared to the original sample, but after 3 months of storage, it showed a shift towards less *Bacteroidaceae*. In comparison, the adsorbate product had a change in relative abundances from day 0 but no changes were observed after 3 months.

These observations were reflected in the Faith diversity index where the adsorbate product at time point 0 showed a reduction (*p* = 0.01) that was not observed in the lyophilized product (*p* = 0.20) compared to the original pool. After 3 months of storage, both formulations have shown a reduction in alpha diversity (adsorbate *p* = 0.02; lyophilized *p* = 0.01). On the other hand, focusing on the Pielou index, we did not observe any significant change in any of the formulations just after preparation (adsorbate *p* = 0.08; lyophilized *p* = 0.84), but after 3 months, there was a reduction only in the adsorbate evenness index (adsorbate *p* = 0.03; lyophilized *p* = 0.50). Analyzing Bray–Curtis dissimilarity distances at the feature level (**Supplementary Figure S2**), both formulations were separately distributed on their own clusters (ADONIS *p* = 0.019), but no differences were observed in terms of time of storage.

### Macroscopic and Humidity Analysis

The capsule morphology was stable during the storage independently of temperature conditions and no odor was detected in lyophilized or adsorbate formulations. A humidity study for the adsorbate formulation after direct encapsulation showed 27.83% (SD 0.98) of water content in capsules versus 9.76% (SD 0.68) if encapsulation was preceded by a desiccation with Silica gel.

### Viability Analysis of Encapsulated Adsorbate Formulation Using Magnesium Stearate or Not at 4°C up to 6 Months

**Figure 5:**
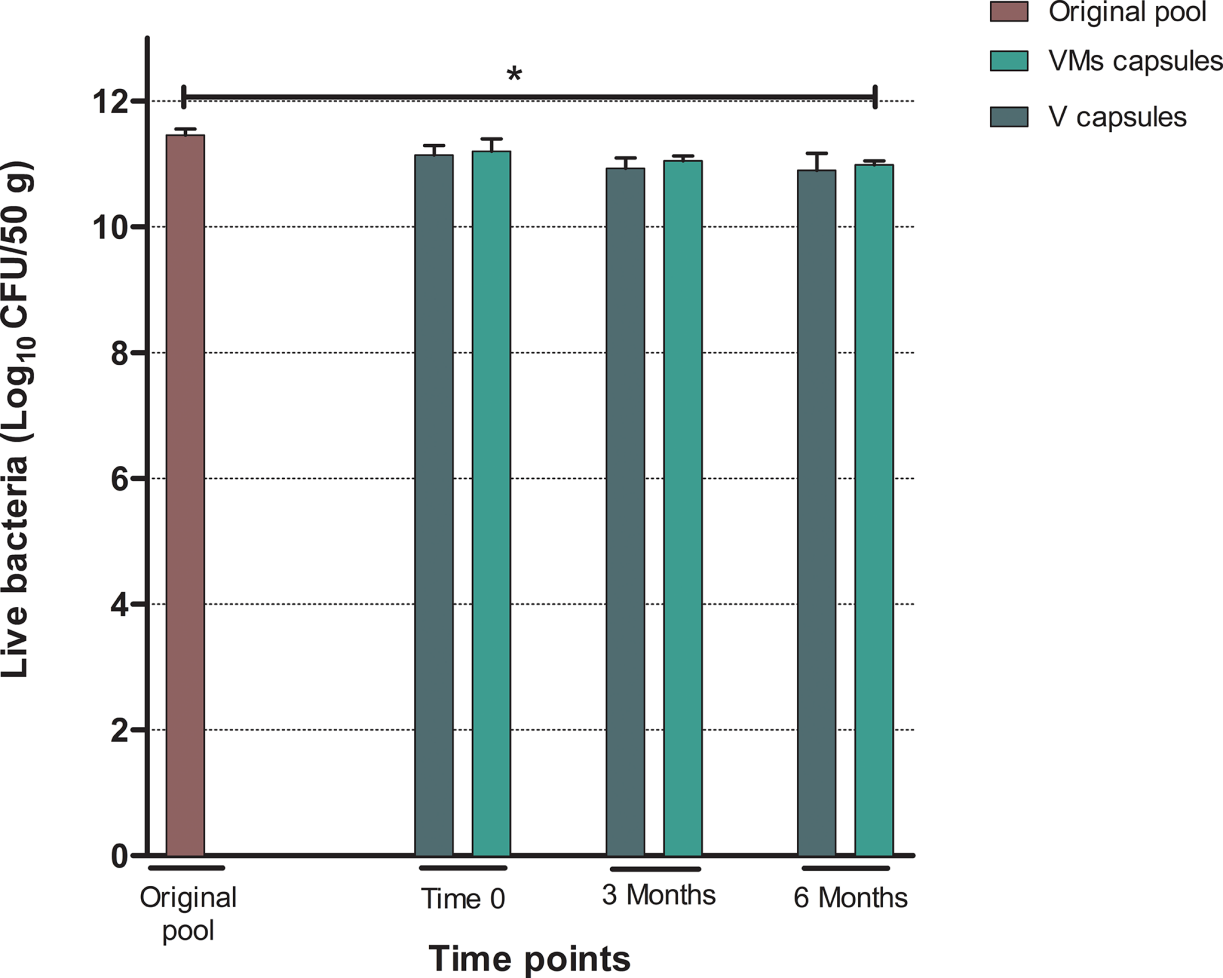
Results from flow cytometry analysis corresponding to the number of viable bacteria expressed as the mean of Log_10_ CFU/50 g of feces (SD). VM capsules: adsorbate capsules with Vivapur-101^®^ in combination with magnesium stearate. V capsules: adsorbate capsules with Vivapur-101^®^ only. Statistical significance *<0.05.

Comparisons of bacterial viability in formulations were made using the mean of original pools as control. Results from flow cytometry (**Table 2** and **Figure 5**) did not show significant differences in viable bacteria at the time of capsule production (V *p* = 0.12; VMs *p* = 0.25) or at 3 months of storage between any of the formulations and the original pool (V *p* = 0.07; VMs *p* = 0.05). At month 6, we observed a slight reduction in the number of viable bacteria in both formulations that achieved significance in VM capsules (*p* = 0.02) but not in V capsules (*p* = 0.06). The results from quantitative bacterial culture supported the results from flow cytometry (**Supplementary Data 2**).

**Table 2:** Results from flow cytometry of Vivapur-101^®^ (V) or Vivapur-101^®^+magnesium stearate (VM) adsorbate capsules.

	Pool	V capsules	*p*	VMs capsules	*p*
*T* = 0	11.46 (0.09)	11.14 (0.15)	*p* = 0.12*	11.20 (0.20)	*p* = 0.25**
*T* = 3 months	NA	10.93 (0.16)	*p* = 0.07*	11.05 (0.08)	*p* = 0.05**
*T* = 6 months	NA	10.90 (0.27)	*p* = 0.06*	10.98 (0.06)	*p* = 0.02**

*Compared to pool results. **Compared to pool results.

Data are presented as the mean of live bacteria (Log_10_ CFU/50 g of feces) from replicates and standard deviation (SD).

NA, Non Applicable.

**Figure 6:**
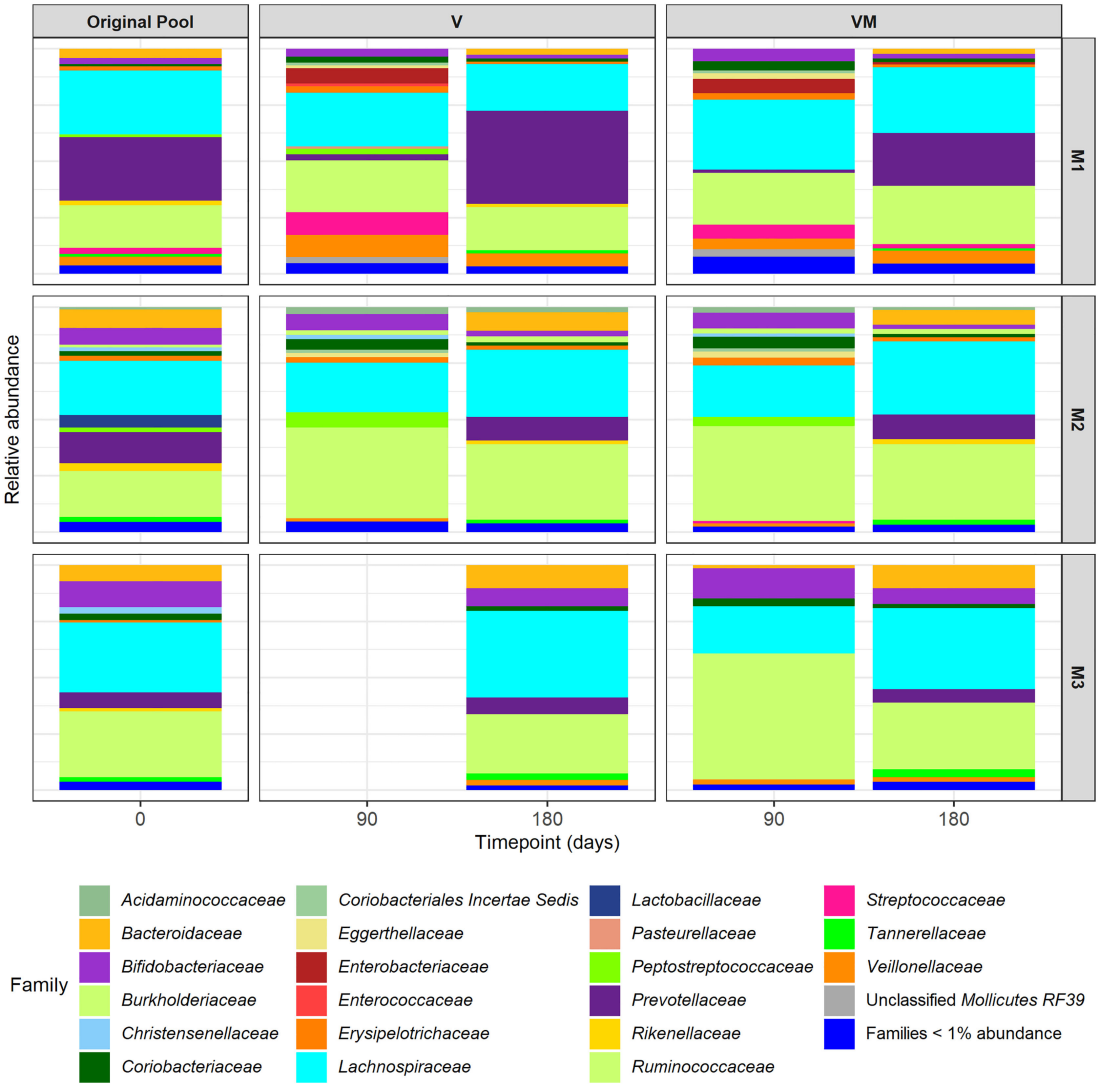
Relative abundances of bacterial taxonomical composition at the family level of original pool and capsules using Vivapur-101^®^ (V) or Vivapur-101^®^+magnesium stearate (VM) of the three experiment samples (M1, M2, and M3). The analysis was performed at 3 months (90 days) and 6 months (180 days) in the 3 individual samples analyzed. The M3 V capsules at 3 months were excluded due to low sequence quality.

The genomic analysis of the product (**Figure 6**) showed a greater loss of anaerobic bacteria over time. However, some well-known families of anaerobic bacteria that are characteristically found in the gut microbiota of healthy individuals were present in the samples up to 6 months. These genera comprised *Bifidobacteriaceae, Ruminococcaceae, Lachnospiracea, Prevotellaceae*, and *Bacteroidaceae*. On the other hand, some bacterial families such *Streptococcaceae* and *Rikenellaceae* disappeared at the final time point.

In addition, the alpha diversity Faith index did not significantly change between the original pool (mean 8.88, SD 0.67) and the adsorbate capsules after 3 months (V *p* = 0.33; VMs *p* = 0.37) or after 6 months (V *p* = 0.92; VMs *p* = 0.45). The Pielou index was slightly reduced between the original pool (mean 0.93, SD 0.02) and the adsorbate capsules after 3 months (V *p* = 0.72; VMs *p* = 0.66), which was closer to significance after 6 months (V *p* = 0.06; VMs *p* = 0.06). Observing Bray–Curtis dissimilarity distances at the feature level (**Supplementary Figure S3**), we identified that samples were not clustered by the excipient used or by time of storage, but they were clustered by the variability of each replicate (ADONIS *p* = 0.001).

## Discussion

FMT has become a first-line treatment for recurrent *C. difficile* infection (McDonald et al., 2018; Mullish et al., 2018; Cammarota et al., 2019; Johnson et al., 2021; van Prehn et al., 2021), and its administration with oral capsules has proved to be as effective as traditional invasive methods (Reigadas et al., 2020). This strategy has several advantages: patients can take the FMT in an ambulatory manner, endoscopic procedures can be avoided, and hospitals will be able to save money (Kao et al., 2017; Reygner et al., 2020). The main challenge of oral formulations is to maintain not only bacterial viability but also its diversity in the minimum number of capsules to keep the functionality of gut microbiota once introduced in the new host. In this study, we compared a previously described encapsulated lyophilized formulation (Staley et al., 2017) with a new encapsulated formulation based on an adsorbate that could potentially be an alternative way for oral FMT administration.

From the first step, our analysis showed that at 4°C, both formulations maintained the bacterial viability for at least 3 months according to flow cytometry results. Despite the limitation of bacterial culture due to the large number of unculturable bacteria in feces, the results from quantitative culture supported the results from flow cytometry. In terms of microbial composition, lyophilized formulation maintained the relative abundances of most bacterial families present in the original sample. This included Bacteroidaceae, one of the key families in healthy gut microbiota (Rinninella et al., 2019), which was not represented in the adsorbate formulation from the beginning of the process. At this point, we hypothesize that the mixing step, performed under aerobic conditions, in the adsorbate formulation procedure exposes more bacteria to oxygen than in the lyophilization procedure. Nevertheless, this step could be optimized by performing the whole process under anaerobic conditions.

Analyzing the results of the second experiment, we observed that the addition of magnesium stearate in the adsorbate formulation did not represent a change in the encapsulation process, although it could imply a reduction of bacterial viability after 6 months. In microbial composition analysis, the relative abundance of other important genera including *Bifidobacteriaceae, Lachnospiraceae*, and *Ruminococcaceae* was maintained after 6 months of storage at 4°C compared to original pools. These families have been associated with a healthy status and the maintenance of gut mucosal health participating in the development of the immune system and the control of inflammatory processes, preventing pathogen colonization or the production of vitamins and metabolites such as short-chain fatty acids (La Rosa et al., 2019; Pittayanon et al., 2019; Rinninella et al., 2019; Vacca et al., 2020; Duranti et al., 2021).

The advantages of lyophilized capsules include the low final volume that reduces the number of capsules per treatment, less odor than frozen products, and stability at 4°C, avoiding the necessity of ultralow-temperature storage (Jiang et al., 2018). However, this is a costly procedure, the standardization of the process is a challenge, and we also found some difficulties in the encapsulation process, described also by other groups (Staley et al., 2017). The adsorbate formulation is a powder that has practical advantages regarding its processing compared to lyophilization. The manufacturing process is faster, does not consume energy, is significantly cheaper since the excipients have a low acquisition cost, and has no odor, and its organoleptic properties make the encapsulation process easier. These characteristics make this new formulation potentially incorporated into industrial processes, expanding FMT accessibility. Additionally, this study showed competitive results in bacterial viability and the stability of microbial composition after 6 months of storage at 4°C, which facilitates its transportation and storage. In contrast, previously described oral capsules for FMT (Youngster et al., 2016; Staley et al., 2017; Reigadas et al., 2020; Reygner et al., 2020) require frozen steps to produce it or maintain stability of the product, hindering the facilities where this product can be available.

Our study has several limitations. First, our results are subject to unrecognized bias because the number of samples was small, and feces have an inherent inter-sample variability. Furthermore, our genomic analysis has a limited resolution and lower sensitivity compared to metagenomic data in feces composition.

In conclusion, the adsorbate formulation performed using microcrystalline cellulose as the main excipient seemed to have a stabilizing effect in gut microbiota, maintaining bacteria viability and preserving its diversity. In the future, it is necessary to improve the early management of the fecal material to reduce the loss of anaerobic bacteria as well as during the mixing process of the adsorbent formulation and to test the encapsulated adsorbent formulation for recurrent CDI treatment.

## Data Availability Statement

The original contributions presented in the study are publicly available. This data can be found here: SRR19025779 - SRR19025792.

## Author Contributions

AA, JS-N, and AS: conceptualization. AA, ER, AR, AV, and JS-N: methodology. AA, ER, AR, and AV: data analysis. AA: writing—original draft preparation. AA, ER, AV, CC-P, VR, JS-N, and AS: writing—review and editing. All authors reviewed the results and approved the final version of the manuscript.

## Funding

This study has been funded by Instituto de Salud Carlos III through the project “PI16/01023” (Co-funded by European Regional Development Fund “Investing in your future”).

## Conflict of Interest

The authors declare that the research was conducted in the absence of any commercial or financial relationships that could be construed as a potential conflict of interest.

## Publisher’s Note

All claims expressed in this article are solely those of the authors and do not necessarily represent those of their affiliated organizations, or those of the publisher, the editors and the reviewers. Any product that may be evaluated in this article, or claim that may be made by its manufacturer, is not guaranteed or endorsed by the publisher.
