# Supplementary figures and images for: New Procedure to Maintain Fecal Microbiota in a Dry Matrix Ready to Encapsulate
Source: Front Cell Infect Microbiol. 2022 Jun 10;12:899257. doi: 10.3389/fcimb.2022.899257 (PMC9226551; doi:10.3389/fcimb.2022.899257)

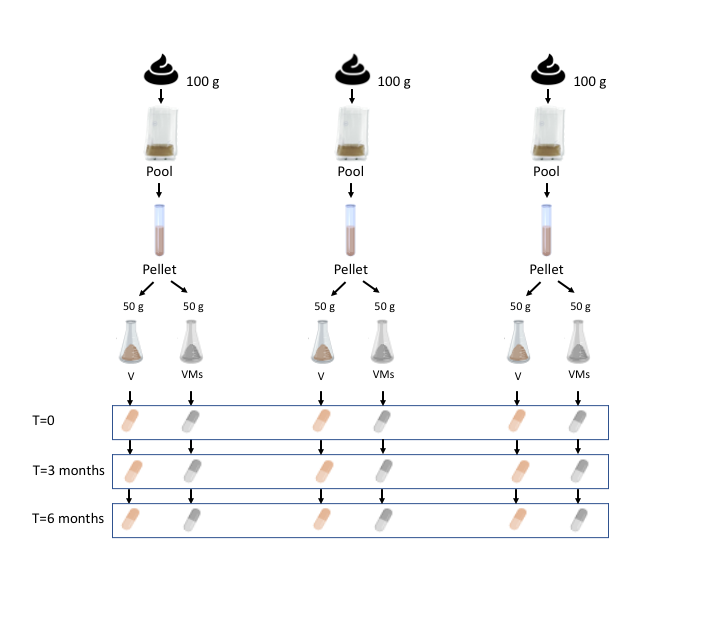

Supplement: Supplementary file 1 [file Image_1.tiff]

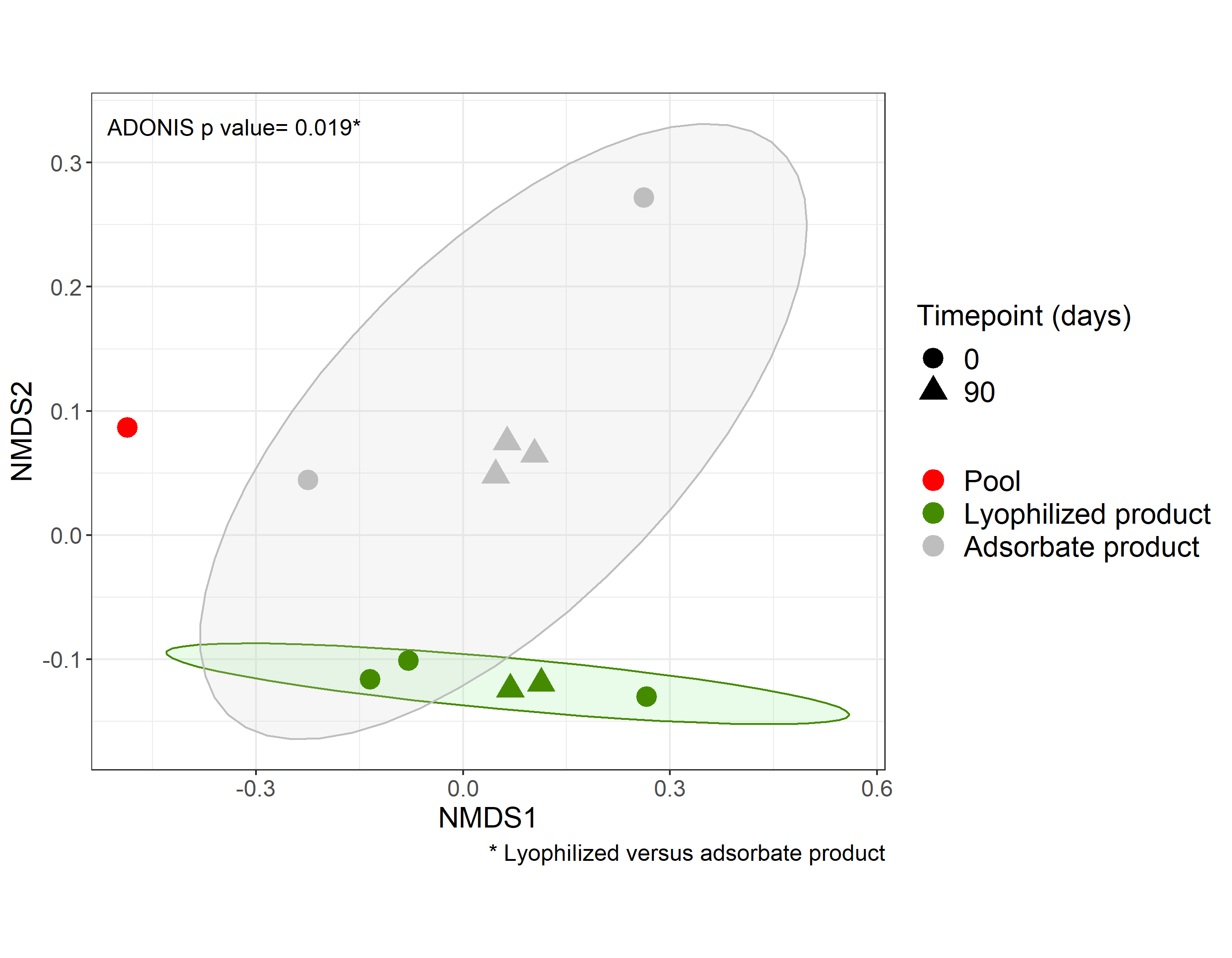

Supplement: Supplementary file 2 [file Image_2.tiff]

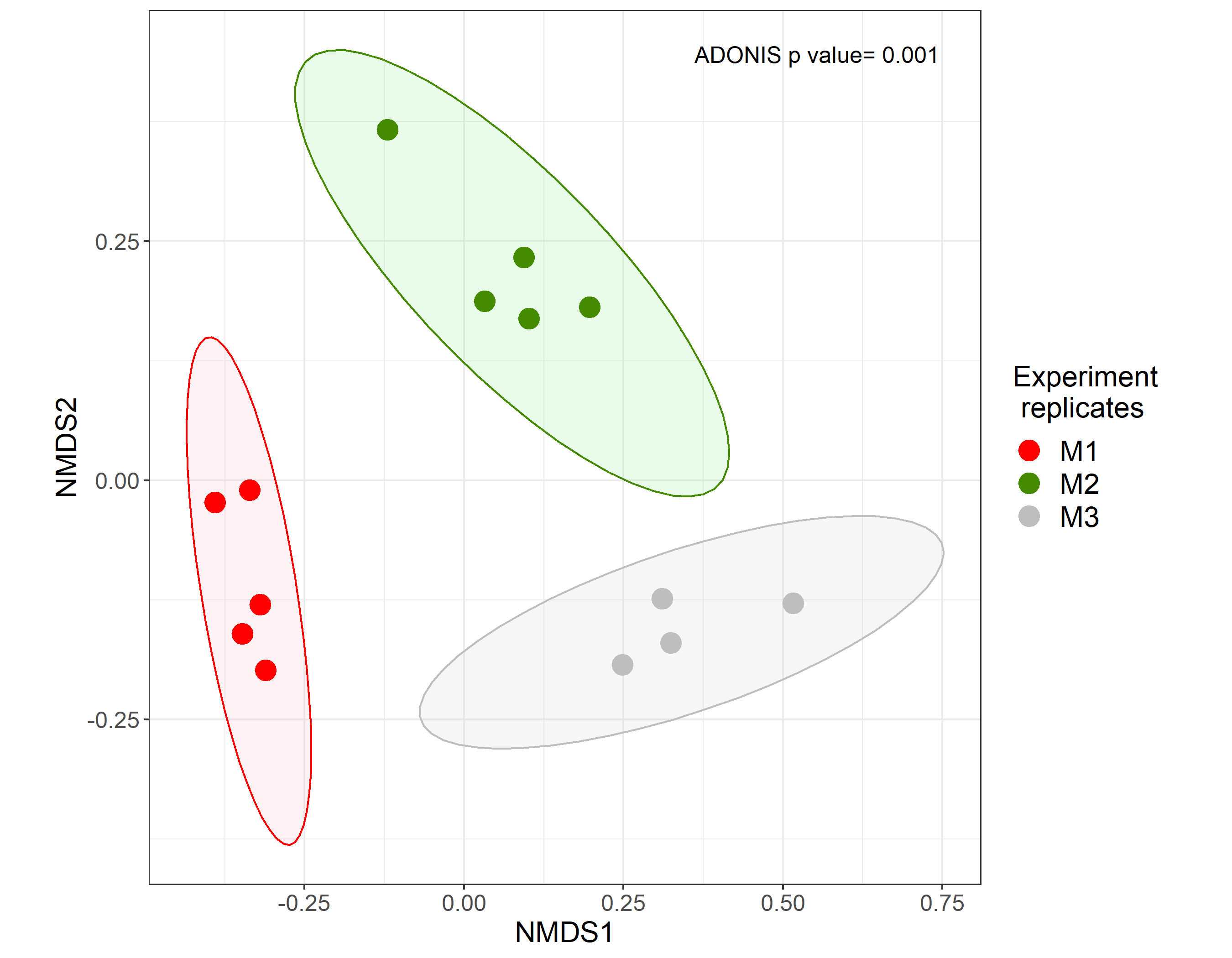

Supplement: Supplementary file 3 [file Image_3.tiff]
